# Negative voltage modulated multi-level resistive switching by using a Cr/BaTiO_x_/TiN structure and quantum conductance through evidence of H_2_O_2_ sensing mechanism

**DOI:** 10.1038/s41598-017-05059-9

**Published:** 2017-07-05

**Authors:** Somsubhra Chakrabarti, Sreekanth Ginnaram, Surajit Jana, Zong-Yi Wu, Kanishk Singh, Anisha Roy, Pankaj Kumar, Siddheswar Maikap, Jian-Tai Qiu, Hsin-Ming Cheng, Ling-Na Tsai, Ya-Ling Chang, Rajat Mahapatra, Jer-Ren Yang

**Affiliations:** 1 Thin Film Nano Tech. Lab., Department of Electronics Engineering, Chang Gung University, 259 Wen-Hwa 1st Rd., Kwei-Shan, Tao-Yuan 33302 Taiwan; 2 Division of Gyn-Oncology, Department of Obs/Gyn, Chang Gung Memorial Hospital (CGMH), Tao-Yuan, 33302 Taiwan; 3 Material and Chemical Research Laboratories (MRL), Industrial Technology Research Institute (ITRI), Hsinchu, 310 Taiwan; 4 Department of Materials Science and Engineering, National Taiwan University, Taipei, 106 Taiwan; 5 Department of Electronics and Communications Engineering, National Institute of Technology (NIT), Durgapur, 713209 India

## Abstract

Negative voltage modulated multi-level resistive switching with quantum conductance during staircase-type RESET and its transport characteristics in Cr/BaTiO_x_/TiN structure have been investigated for the first time. The as-deposited amorphous BaTiO_x_ film has been confirmed by high-resolution transmission electron microscopy. X-ray photo-electron spectroscopy shows different oxidation states of Ba in the switching material, which is responsible for tunable more than 10 resistance states by varying negative stop voltage owing to slow decay value of RESET slope (217.39 mV/decade). Quantum conductance phenomenon has been observed in staircase RESET cycle of the memory devices. By inspecting the oxidation states of Ba^+^ and Ba^2+^ through measuring H_2_O_2_ with a low concentration of 1 nM in electrolyte/BaTiO_x_/SiO_2_/p-Si structure, the switching mechanism of each HRS level as well as the multi-level phenomenon has been explained by gradual dissolution of oxygen vacancy filament. Along with negative stop voltage modulated multi-level, current compliance dependent multi-level has also been demonstrated and resistance ratio up to 2000 has been achieved even for a thin (<5 nm) switching material. By considering oxidation-reduction of the conducting filaments, the current-voltage switching curve has been simulated as well. Hence, multi-level resistive switching of Cr/BaTiO_x_/TiN structure implies the promising applications in high dense, multistate non-volatile memories in near future.

## Introduction

Recently, resistive random access memory (RRAM) has attracted much attention because of its potential to replace three-dimensional (3-D) flash memory in future^1–3^. In this regard, many research groups have proposed different switching materials like binary metal oxides and transition metal oxide^4^ such as Ta_2_O_5_^3, 5^, TiO_2_^6^, HfO_2_^7^, etc. Among them, the perovskite oxides such as SrTiO_3_^8, 9^, SrZrO_3_^10^ and BaTiO_3_^11, 12^ have drew enormous attention towards the application since last decade. Importantly, BaTiO_3_ has high dielectric constant of 100–600^13^ and large band gap of 3.42 eV^14^, which is one of the potential resistive switching materials. The demand of high density data storage can effectively be achieved by multi-level resistive memory cell. Multi-level resistive switching operation of five resistance states using different materials in ITO/RGO/ITO^15^ and TiN/Ta_2_O_5_/Pt^16^ structures have been demonstrated. Several research groups have reported multi-level switching operation with multiple states for brain-inspired neuromorphic applications^17, 18^. However there is no report on tunable multi-level resistive switching characteristics of the BaTiO_x_ switching material in Cr/BaTiO_x_/TiN structure and its transport mechanism in each level is not reported yet. In addition, quantum conductance occurs due to movement of the oxygen vacancies^19^ when the contact point of filament is reduced to atomic scale. Nowadays, this phenomenon is at the center of attraction due to its possible application in multi-level and neuromorphic resistive memory^20–22^. Chen *et al*.^19^ have reported anion migration based quantum conductance in Ti/Ta_2_O_5_/Pt structure. Younis *et al*.^20^ have reported voltage sweep rate dependent quantum conductance in Au/SnO_2_–CeO_2_/FTO structure. In this report, negative voltage dependent quantum conductance in a novel Cr/BaTiO_3_/TiN structure has been reported for the first time. The oxidation-reduction (redox) process and change in oxidation state of Ba is responsible for multi-level and quantum conductance phenomenon. The staircase oxidation of Ba in switching material, BaTiO_3_ is justified by the sensing of hydrogen-peroxide (H_2_O_2_) in electrolyte-insulator-semiconductor (EIS) structure, which is also completely novel approach presented in this paper.

The negative stop voltage modulated multi-level resistive switching in Cr/BaTiO_x_/TiN structure is observed due to gradual dissolution of oxygen vacancy filament. Quantum conductance is observed at staircase RESET in which the experimental result fits very well with the simulated curve. The X-ray photoelectron spectroscopy shows oxidation states of Ba and Ti. The oxidation-reduction of Ba is responsible for resistive switching mechanism and multi-level resistance states are due to more generation of Ba^2+^ ions under staircase RESET. The rate of dissolution of filament with negative stop voltage i.e. the increase of high resistance state (HRS) with negative stop voltage is uniform and controllable moderate value of 217.39 mV/decade is obtained for lower thickness (2.5 nm) of BaTiO_x_. The devices with 0.4 × 0.4 μm^2^ size exhibit better resistive switching than devices with 4 × 4 μm^2^ size. In both positive and negative voltage cycles of low resistance state (LRS), the Ohmic conduction is observed in low field where as the hopping conduction is observed in high field for both devices. In HRS, Poole-Frenkel and hopping conduction are observed in moderate and high field, respectively. Moreover, the Fowler-Nordheim tunneling is observed in very high negative voltage. The switching mechanism including multi-level operation and quantum conductance in staircase RESET is explained through evidence of H_2_O_2_ sensing with concentration of 1 nM to 1000 nM in electrolyte/BaTiO_x_/SiO_2_/p-Si structure. In addition, the BaTiO_x_ membrane shows good sensitivity of 48 mV/pH. The devices also exhibit high resistance ratio of 2000, high speed program/erase endurance of more than 10^7^ cycles with 100 ns pulse width and 3 hours data retention at 85 °C. This unique presentation of switching mechanism through H_2_O_2_ sensing shows a path towards combination of resistive memory and bio-sensor.

## Results and Discussion

**Figure 1:**
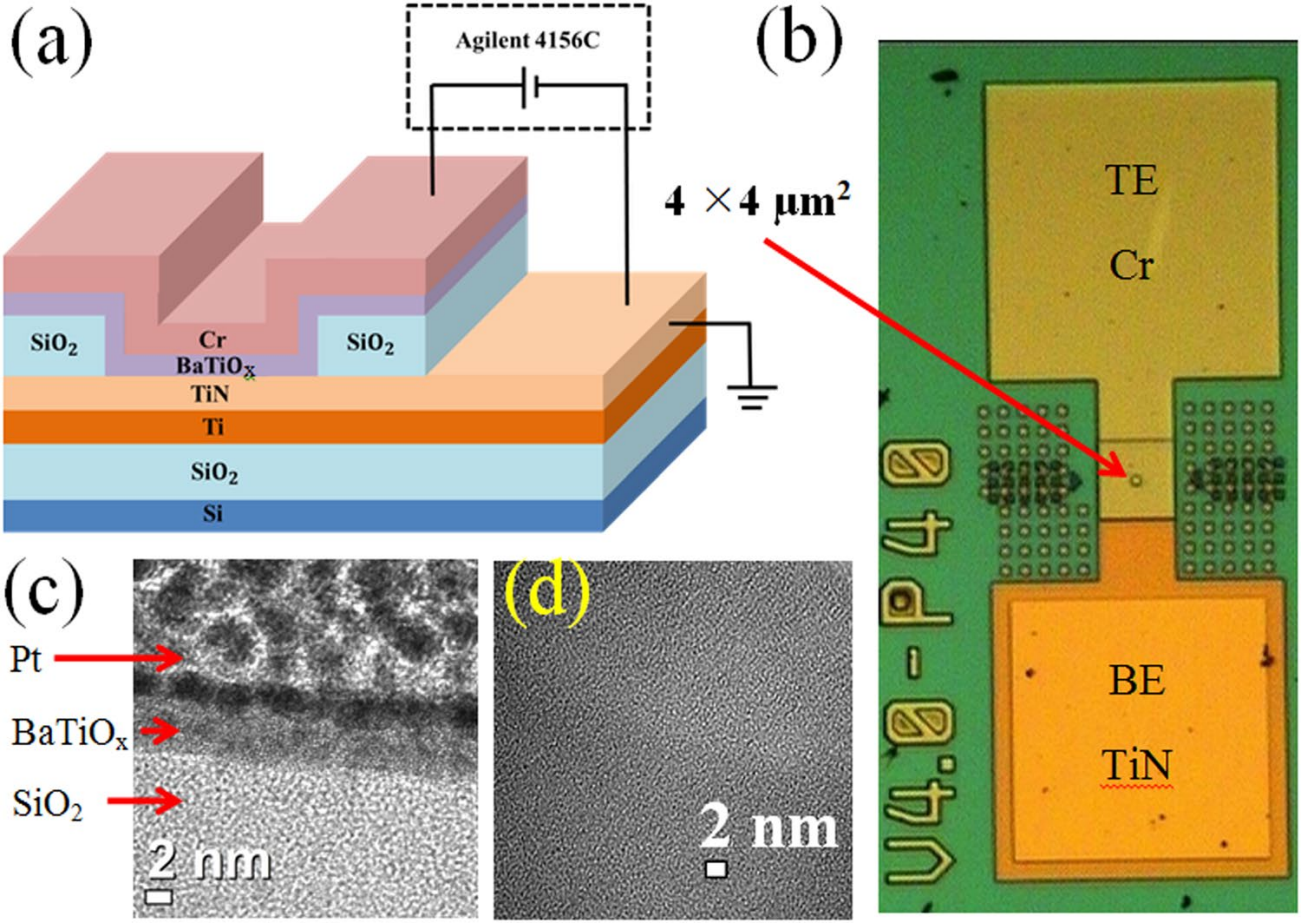
(**a**) Schematic view of the device and measurement setup with Agilent 4156C. (**b**) Optical microscope (OM) image of a device. (**c**) Cross-sectional HRTEM image confirms the amorphous BaTiO_x_ layer. (**d**) Plane-view TEM image confirms amorphous BaTiO_x_ SM also.

**Figure 2:**
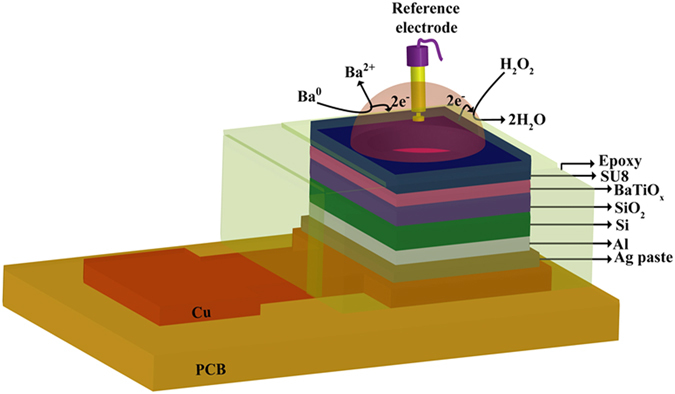
Schematic view of pH and H_2_O_2_ sensing using BaTiO_x_ membrane in EIS structure.

A schematic view of resistive switching memory device is shown in Fig. 1a. An optical microscope image with a size of 4 × 4 µm^2^ is also shown in Fig. 1b. Figure 1c shows the cross-sectional transmission electron microscopy (TEM) image with 5 nm-thick BaTiO_x_ layer. Plane-view TEM image shows amorphous BaTiO_x_ film (Fig. 1d). Detection of pH and H_2_O_2_ is performed by using electrolyte/BaTiO_x_/SiO_2_/p-Si structure, which is shown in Fig. 2 schematically.

### XPS characteristics

**Figure 3:**
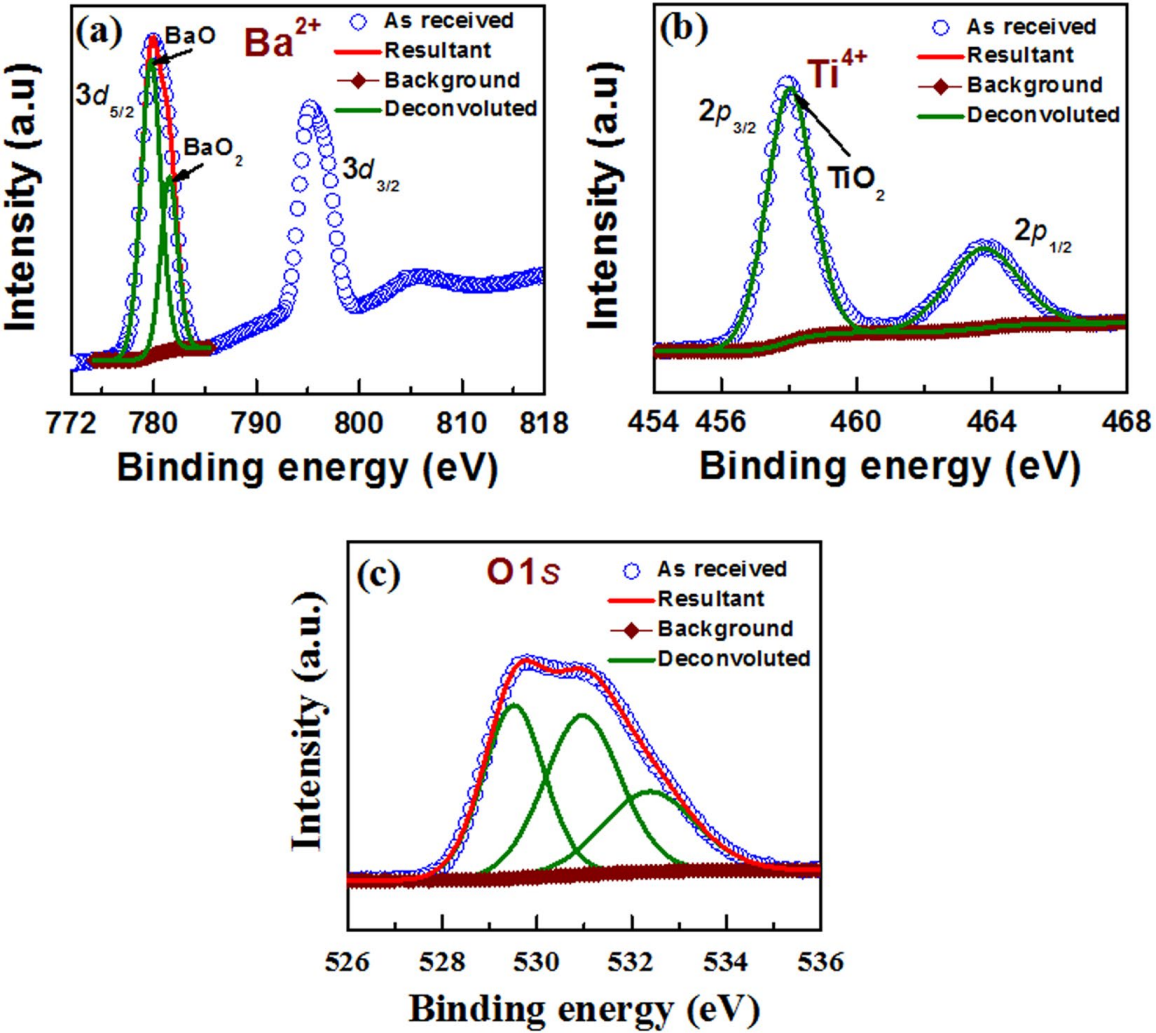
XPS characteristics of (**a**) Ba*3d*, (**b**) Ti*2p*, and (**c**) O*1s* spectra from the BaTiO_x_ SM.

Figure 3 shows the XPS analysis of the switching materials (SMs). The doublet spectra of Ba3*d*_*5*/*2*_ and Ba3*d*_*3*/*2*_ are fitted at 780 eV and 795.2 eV, respectively (Fig. 3a). Forster *et al*.^23^ have reported similar binding energy peaks at 779.6 eV and 795 eV for the Ba3*d*_*5*/*2*_ and Ba3*d*_*3*/*2*_ peaks, respectively. The Ba 3*d*_*5*/*2*_ peak consists of two components, which correspond to BaO at 779.8 eV and BaO_2_ at 781.6 eV. Droubay *et al*.^24^ have reported similar observation where the peak binding energy at 775.5 eV corresponds to BaO and the peak binding energy at 779 eV corresponds to BaO_2_. For both BaO and BaO_2_ species, Ba has ionic state of 2+. The oxidation state of oxygen (O) in BaO is 2- and it is 1- for BaO_2_. Figure 3b shows XPS of Ti doublet spectra at 458 eV and 464 eV, which corresponds to Ti2*p*_*3*/*2*_ and Ti2*p*_*1*/*2*_ peaks. These values are close to our previous reported values, 458.8 eV for Ti2*p*_*3*/*2*_ peak and 464.4 eV for Ti2*p*_*5*/*2*_ peak^25^. These Ti2*p* peaks correspond to TiO_2_, where the oxidation state of Ti is 4+^26^. In addition, there is no oxidation state of Ti for 3+ or 2+, where Ti2*p*_*3*/*2*_ peaks are centered at 457.6 eV and 456.4 eV, respectively. The O*1s* spectrum is de-convoluted into three peaks at 529.5 eV, 531 eV and 532.4 eV, where first two peaks correspond to BaO at 529.5 eV and BaO_2_ at 531 eV. These values are close to the reported values of 528.9 eV for BaO and 531.1 eV for BaO_2_^27^. Hashimoto *et al*.^28^ have reported that the energy peak centered at 528.9 eV is owing to TiO_2_. The energy peak entered at 532.4 eV is due to hydroxide (OH) groups on BaTiO_x_ surface. Chu *et al*.^29^ have reported that O*1s* peak centered at 532.9 eV is hydroxide groups on TiO_2_ nanotube’s surface. From the XPS data the composition of switching material is BaTiO_x_ (1.98 < x < 3), which shows less oxygen or oxygen vacancies in SM. The O*1s* peak located at 531 eV may be due to possible oxygen deficiencies in TiO_2_ film, which is similar to the reported peak binding energy of 531.3 eV^29^. By considering Gibbs free energy at 300 K (−1114.1 kJ/mol for BaO and −887.62 kJ/mol for TiO_2_^30^), there are strong Ti-O bonds or stoichiometric TiO_2_ than the Ba-O bonds or BaO_2_, i.e., BaO_x_. To check thermal stability, the BaTiO_x_ films were annealed at 450 °C, 600 °C, and 750 °C. There is negligible change of Ba oxidation state and the composition is stable up to 600 °C. The details of XPS analysis at high temperature are given in the supplementary information (Fig. S1).This will lead to good resistive switching memory characteristics as well as H_2_O_2_ sensing, which have been explained below.

### Negative voltage modulated multi-level and transport mechanism

**Figure 4:**
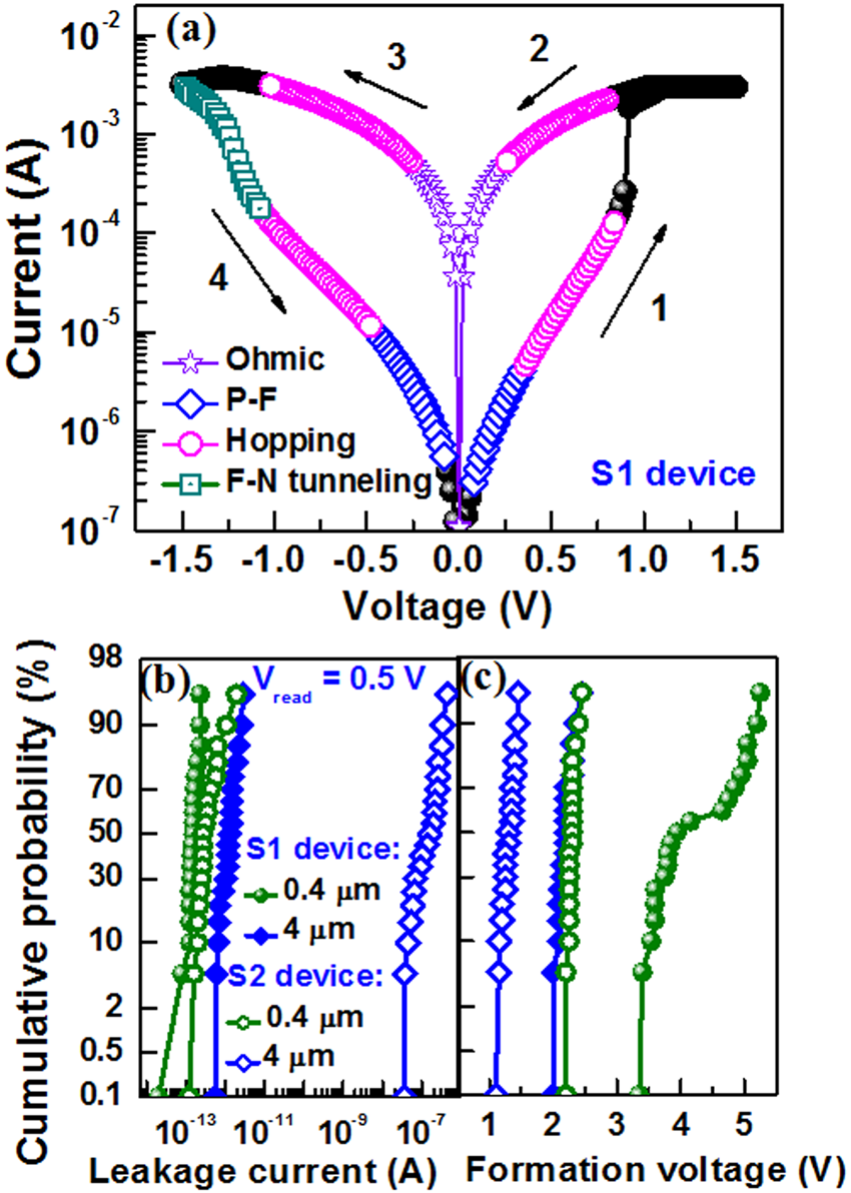
(**a**) Typical bipolar resistive switching characteristics of the S1 devices. Cumulative probability of (**b**) leakage current and (**c**) formation voltage for randomly measured many S1 and S2 devices.

**Figure 5:**
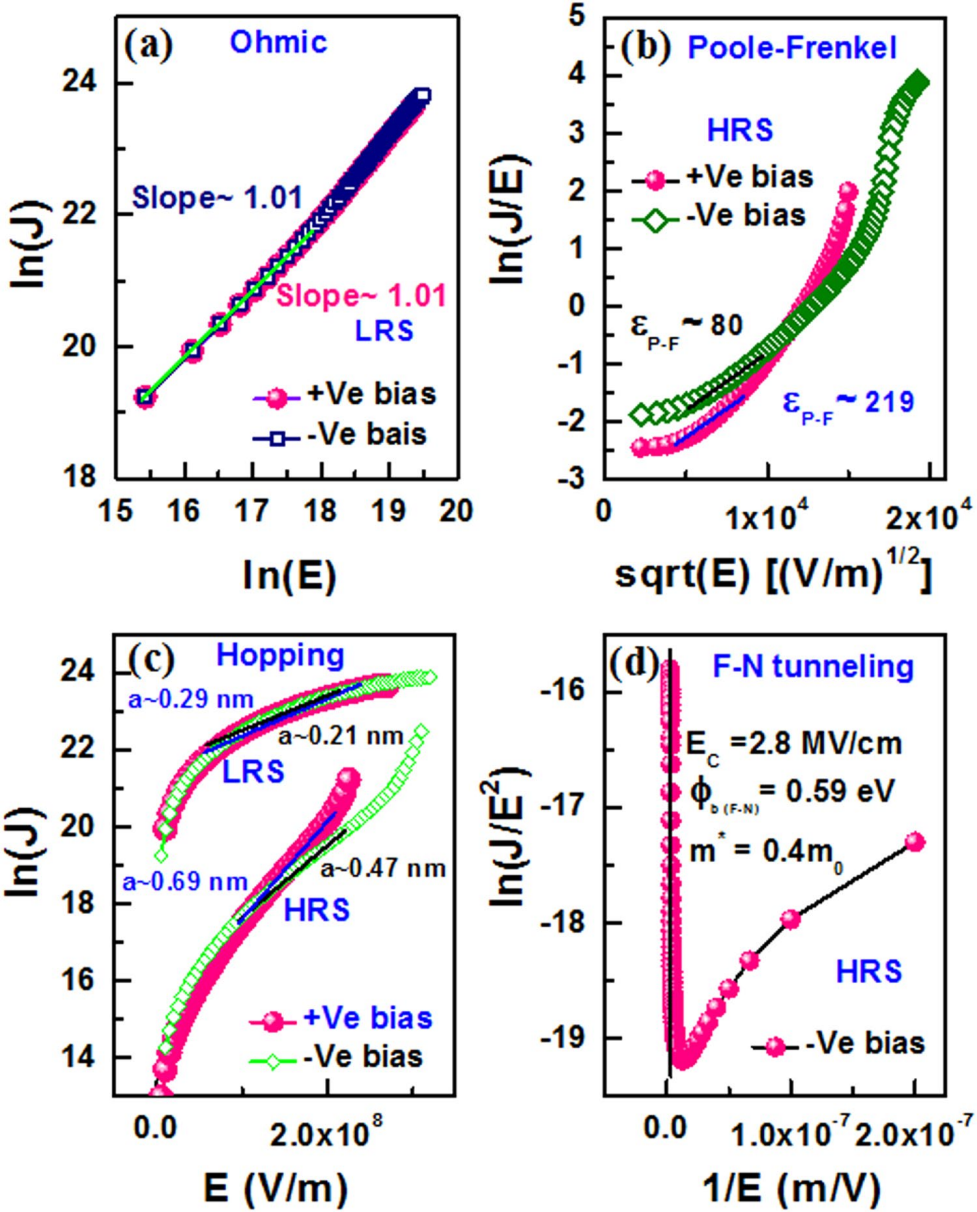
Current transport characteristics of S1 device with a size of 0.4 × 0.4 µm^2^. (**a**) Ohmic conduction, (**b**) Poole-Frenkel (P-F), (**c**) Hopping conduction, and (**d**) Fowler-Nordheim (F-N) tunneling.

Figure 4a shows typical bipolar resistive switching characteristics of the S1 devices with size of 0.4 × 0.4 µm^2^. The SET and RESET voltages are 0.84 V and −1.3 V, respectively. The device is operated under ± 1.5 V. The sweeping bias is shown by arrows 1 to 4. Cumulative distributions of the leakage current and formation voltage are shown in Fig. 4b and c, respectively. Larger (4 × 4 µm^2^) devices of S1 show lower mean value of leakage current than that of the S2 devices (1.347 pA vs. 0.167 µA) while those values for smaller devices (0.4 × 0.4 µm^2^) have the similar trend (0.153 pA vs. 0.434 pA). This indicates that both the larger size devices with thinner SM have higher leakage current owing to more defect paths and shorter lengths^31^. Accordingly, the mean value of formation voltage is lower for the larger (4 × 4 µm^2^) S2 devices than the S1 devices (1.295 V vs. 2.19 V) and those values for the smaller (0.4 × 0.4 µm^2^) devices show a similar trend also (2.287 V vs. 4.207 V). S. Yazdanparast^32^ has reported the reduction of formation voltage by increasing device size for the electrodeposited cuprous oxide as switching material. The standard deviation of formation voltage for the smaller (0.4 × 0.4 µm^2^) S1 devices is higher than the S2 devices (0.673 V vs. 0.06 V). Therefore, the smaller size devices need thinner SM which has benefit of lower operation voltage of <2.287 V with high uniformity as well as scaling further the RRAM device. However, the current transport mechanism is one of the important issues to develop the RRAM device in future. So the I-V curves are fitted with all possible transport mechanisms (Fig. 4a). The LRS currents show Ohmic conduction at low field regions for both positive (+Ve) and negative (−Ve) sides. By plotting ln(J)-ln(E), the slope values are found to be the same 1.01 (Fig. 5a). The HRS current is fitted with Poole-Frenkel (P-F), hopping, and Fowler-Nordheim (F–N) tunneling in sequence of moderate field to high field regions. At higher field, the P-F is fitted (Fig. 5b) and corresponding value of dielectric permittivity is calculated by using equation (1) as given below^33, 34^,

1 $${\varepsilon }_{PF}=\frac{{q}^{3}}{{({k}_{B}T\times {S}_{PF})}^{2}\pi {\varepsilon }_{0}}$$

where q is the electronic charge, k_B_ is the Boltzmann’s constant, T is absolute temperature, ε_0_ is free-space permittivity, and S_PF_ is the slope of the fitted line. The ε_PF_ values are found to be 219 and 80 for both positive and negative bias, respectively and those values are in the range of reported values of 100–600 for BaTiO_3_ film^13^. Oxygen vacancies in SM play a crucial role in the electrical characteristics of the BaTiO_3_ films^35, 36^. At moderate field regions of both HRS and LRS currents, the hopping conduction is observed by fitting ln(J) vs. E curves (Fig. 5c) and corresponding hopping distance (a) is expressed as equation (2)^5, 37^,

2 $$a=\frac{{k}_{B}T}{q}\times {S}_{H}$$

where S_H_ is the slope. The hopping distances at HRS and LRS of +Ve bias are found to be 0.69 nm and 0.29 nm, while those values are 0.47 nm and 0.21 nm for -Ve bias, respectively. P. J. Freud *et al*.^38^ have reported a hopping distance of 0.3 nm for localized charge carrier conduction through the Ni_0.6_Mn_2.4_O_4_ material. In our previous report^34^, the hopping distance of 0.56 nm was reported for IrO_x_/GdO_x_/Al_2_O_3_/TiN resistive switching memory device. The hopping distance at LRS is shorter than the value at HRS owing to thin oxygen-rich layer formation at the Cr/BaTiO_x_ interface. For HRS, the F-N tunneling is observed at high field and the barrier height (Φ_b_) is calculated by plotting ln(J/E^2^) vs. 1/E as represented in equation (3) below^39^,

3 $${\phi }_{b}=\frac{S{{}_{FN}}^{2/3}}{3.6\times {10}^{6}\times {\chi }^{1/3}}$$

where S_FN_ is the slope, m^*^ ( = χ  ×  m_0_) is the effective mass of electron, m_0_ is the rest mass of electron, and the value of χ is considered to be 0.4. The critical field (E_c_) is 2.8 MV/cm (Fig. 5d). The typical value (>2.6MV/cm) of E_c_ is reported for F-N tunneling^40, 41^. The φ_b_ value is cal_c_ulated to be 0.59 eV, which is close to the difference of Cr work function (4.5 eV^42^) and electron affinity of BaTiO_3_ (3.9 eV^36^). Similar conduction mechanism and negative voltage modulated multi-level operation under RESET are also observed for the S2 devices as discussed below.

**Figure 6:**
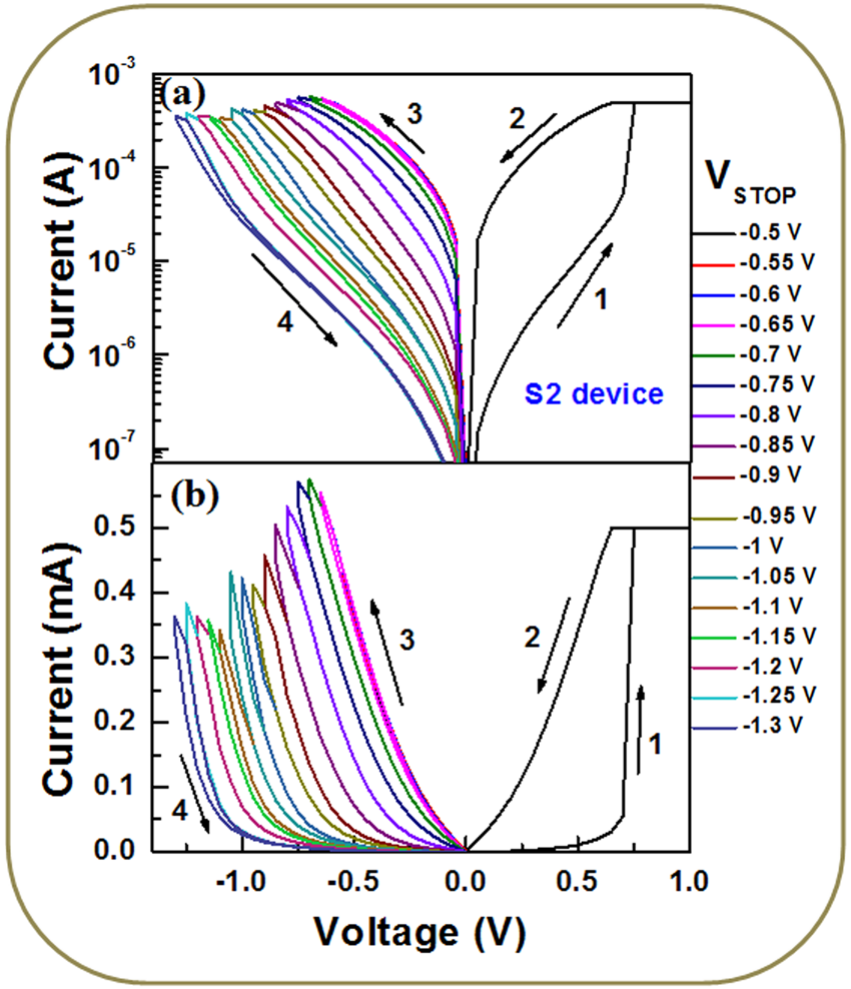
One time SET and staircase RESET by varying negative stop voltage of the S2 devices with a size of 0.4 × 0.4 µm^2^. I-V characteristics using (**a**) log and (**b**) linear scales.

**Figure 7:**
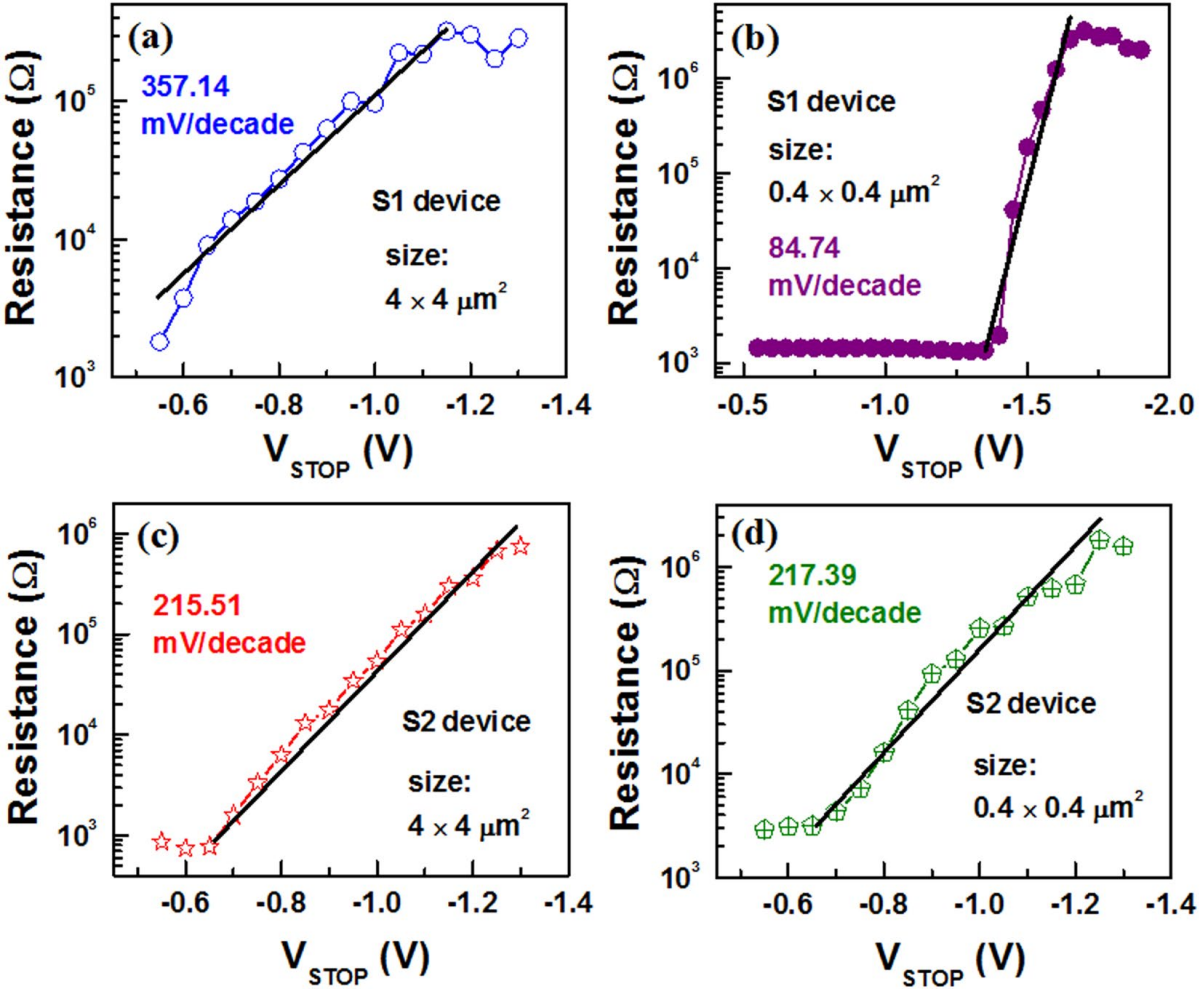
Variation of HRS with negative stop voltage of the S1 devices with (**a**) 4 × 4 µm^2^ and (**b**) 0.4 × 0.4 µm^2^. The S2 devices with (**c**) 4 × 4 µm^2^ and (**d**) 0.4 × 0.4 µm^2^.

**Figure 8:**
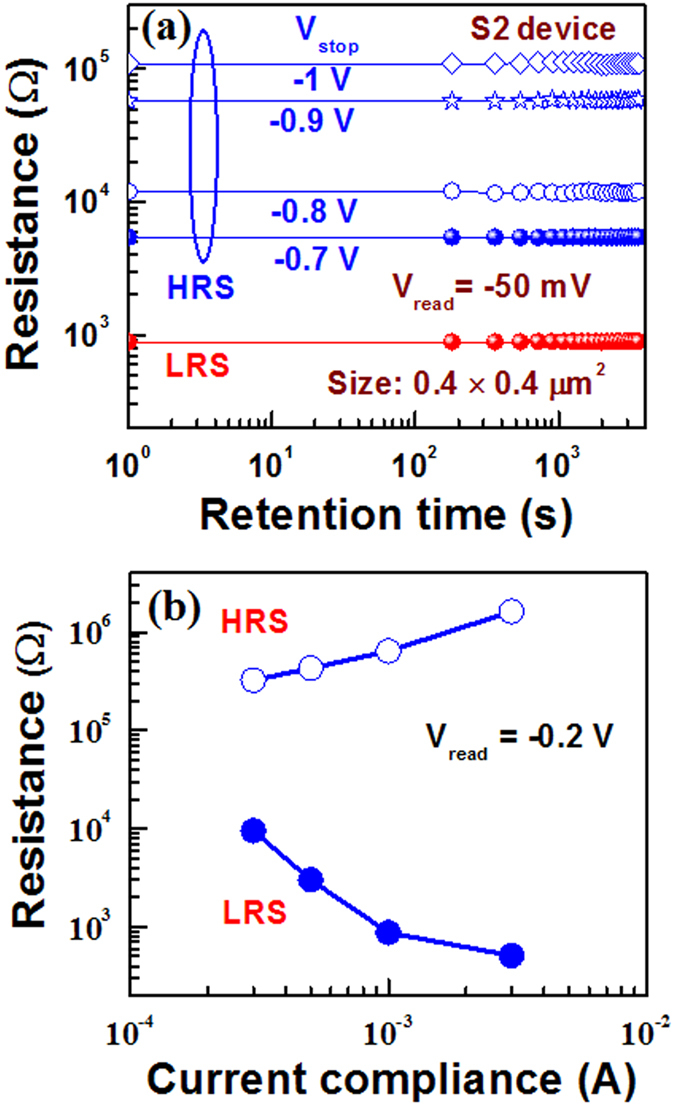
(**a**) Data retention for multi- levels corresponding to stop voltages of −0.7 V, −0.8 V, −0.9 V and −1 V. (**b**) Current compliance dependent multi-level resistance changes in both LRS and HRS.

**Figure 9:**
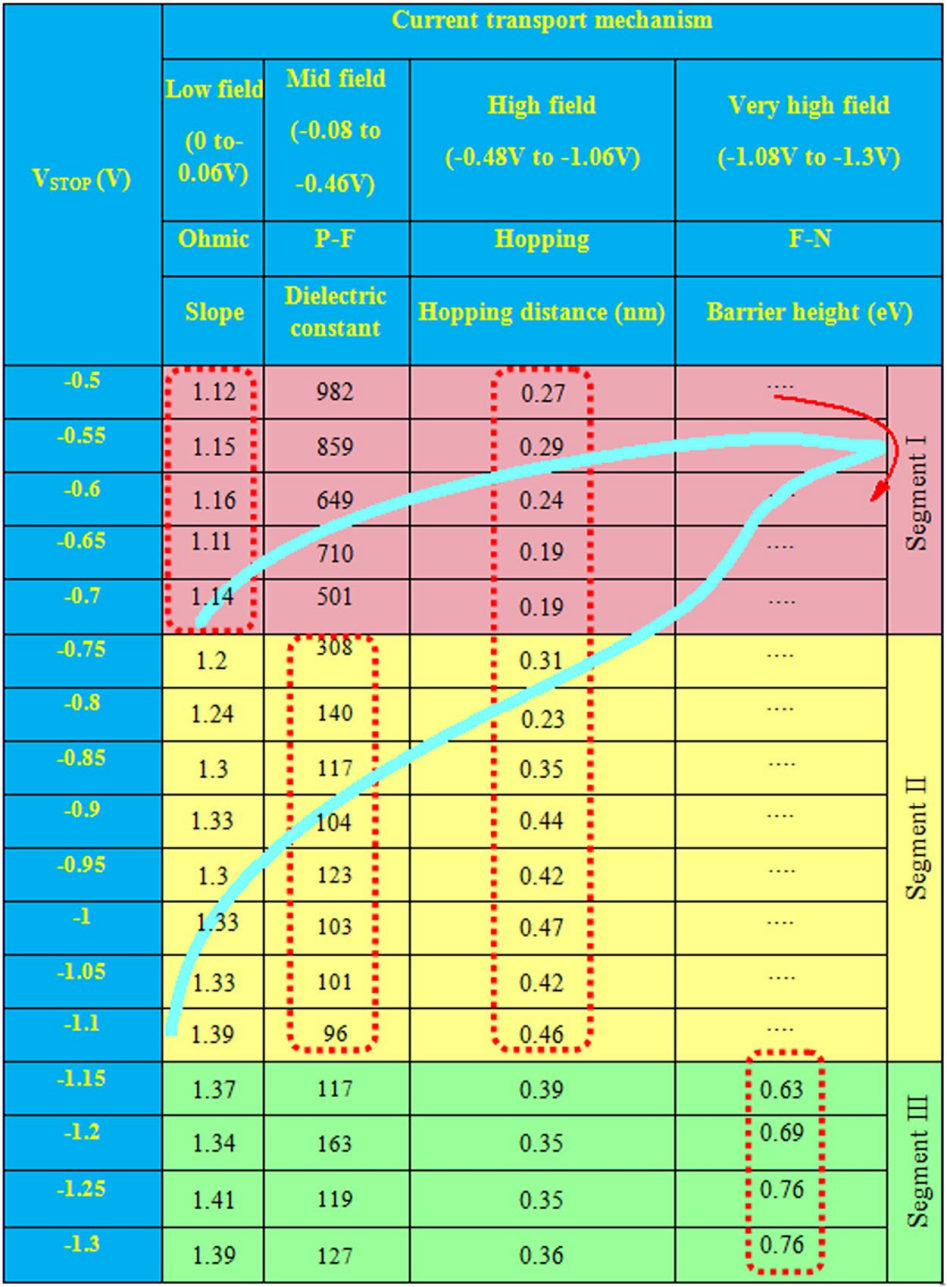
Parameter obtained from current transport characteristics under RESET. Typical I-V curve changing negative voltage of the S2 devices with a size of 0.4 × 0.4 µm^2^.

First, the device sets and the conductance is decreased by increasing value of negative stop voltage (V_STOP_) at RESET (Fig. 6), where I–V curves are plotted in log scale (Fig. 6a) and linear scale (Fig. 6b). Then V_STOP_ value is started −0.5 V and step voltage is −50 mV until complete RESET is achieved at −1.3 V. The current value is read out at −50 mV. The resistance value is increased from 3.15 kΩ (V_STOP_ = −0.5 V) to 1820 kΩ (V_STOP_ = −1.3 V), due to V_STOP_ modulated gradual RESET as well as multi-level of the device is observed. The multi-level operation can easily be realized in resistance vs. V_STOP_ plot for both the S1 and S2 devices (Fig. 7). A numerical parameter is introduced which is ratio of change in V_STOP_ to change in resistance (mV/decade), which is similar to the subthreshold swing (SS) of a metal-oxide-semiconductor filed-effect-transistor (MOSFET). The SS values of the larger (4 × 4 µm^2^) sizes are found to be 375.14 (Fig. 7a) and 215.51 (Fig. 7b) mV/decade, while those values of the smaller (0.4 × 0.4 µm^2^) sizes are found to be 84.74 (Fig. 7c) and 217.39 (Fig. 7d) mV/decade for the S1 and S2 devices, respectively. Higher SS value is needed to design multiple resistance states. From those SS values, it is inferred that larger size device with thicker SM or smaller size device with thinner SM is useful for multi-level operation. On the other hand, smaller device size with thicker SM has larger filament diameter because of hard breakdown of SM. This will have larger value of RESET voltage as compared to the other devices (−1.6 V vs. −1.3 V). Therefore, the S2 devices with smaller size of 0.4 × 0.4 µm^2^ have higher SS value that will give an opportunity for tuning multi-level high resistance states, even the smaller SM thickness of 2.5 nm has been used. Ten states with resistances of approximately 3.2, 4.3, 7.2, 17, 42, 93, 128, 288, 685, and 1700 kΩ are obtained. Typical data retention characteristics for LRS and four HRS at V_STOP_ of −0.7 V, −0.8 V, 0.9 V and −1 V are also shown in Fig. 8a. These resistance states are stable. Similarly, the LRS value is decreased with increasing current compliances from 300 µA to 3 mA (Fig. 8b) because of the increment of filament diameter^43^. The HRS value is also increased with increasing CC owing to generation of more BaO_2_ in BaTiO_x_ SM. A high resistance ratio ranging from 34 to 3200 are obtained with CCs from 300 µA to 3 mA. To explore the transport mechanism further in multi-level operation (Fig. 6), I–V curves have been fitted at different V_STOP_ ranging from −0.5 V to −1.3 V. The fitting parameters are listed in Fig. 9. In the segment I, when the V_STOP_ value is in the range of −0.5 V to −0.7 V, the slope value is 1.12–1.16, which is owing to Ohmic conduction. In segment II, when the V_STOP_ values are from −0.75 V to −1.1 V, P-F emission at mid field region (−0.08 V to −0.46 V) and hopping conduction at high field region (−0.48 V to −1.06 V) are observed. The slope values are >1.2, which indicates no Ohmic conduction at moderate field regions. The ε_PF_ values are found to be 308 to 96 and ‘a’ values are from 0.31 nm to 0.47 nm. When the applied V_STOP_ is in the range from −1.15 V to −1.3 V (segment III), the ε_PF_ values are from 117 to 163, ‘a’ values are from 0.35 nm to 0.40 nm, and the Φ_b_ values are from 0.63 eV to 0.76 eV. Therefore, the transport mechanism has been modulated by values of V_STOP_. By applying bias of more than SET voltage, the oxygen ions (O^2−^) migrate towards Cr TE and leaving oxygen vacancy from TiN BE forming conducting filament (CF) and an oxygen-rich layer at the Cr/BaTiO_x_ interface is also formed. On the other hand, the CF is dissolved in stair-case by applying negative bias of less than RESET voltage. This shows multiple states because of gradual oxidation of CF or generation of more Ba^2+^ ions, which has been explained by quantum conductance through evidence of H_2_O_2_ sensing as follows.

### Quantum conductance and H_2_O_2_ sensing mechanism

**Figure 10:**
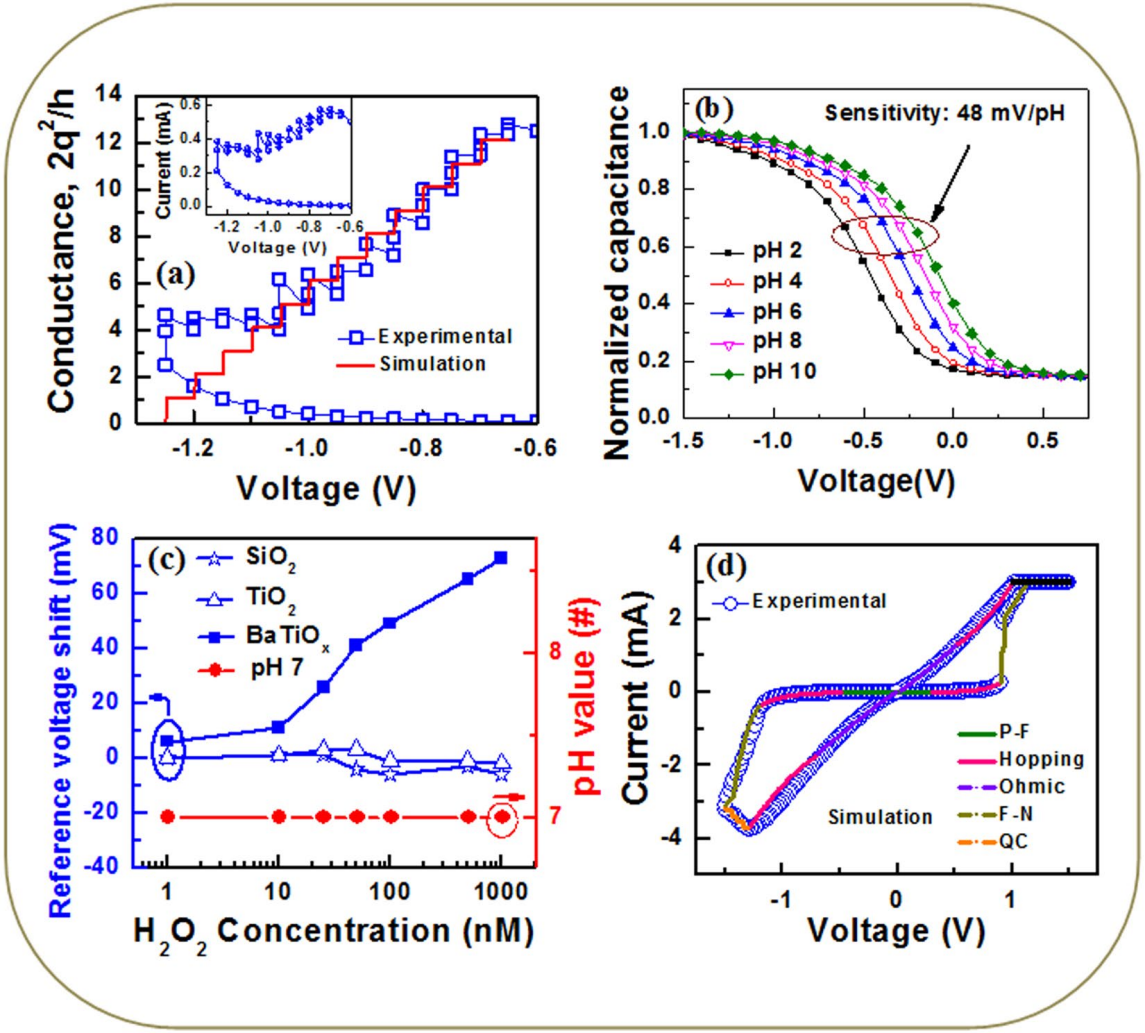
(**a**) Experimental and simulated conductance curve as a function of voltage. Inset shows the I–V of the RESET region. (**b**) C–V characteristics with pH values of 2 to 10. (**c**) Reference voltage shift with H_2_O_2_ concentration from 1 nM to 1000 nM. (**d**) Experimental and simulated I-V curves.

An interesting phenomenon is observed by arranging multi-level I-V curves under gradual RESET of voltage ranging from −0.65 V to −1.05 V (Fig. 6). We can see that the RESET process is consisted of several abrupt current changes, as shown in the inset of Fig. 10a. The conductance (G = I/V) has been calculated in terms of quantum conductance phenomenon, G_0_ ( = 2q^2^/h), where h is Plank’s constant (h = 6.62 × 10^−34^ m^2^ kg/s). Figure 10a shows conductance vs. negative voltage which is changed in a staircase fashion. The values of conductance at −0.8 V, −0.85 V, −0.9 V and −0.95 V are found to be 10G_0_, 9G_0_, 7.5G_0_, and 6.5G_0_, respectively. So the conductance values are integer or half-integer multiple of G_0_^21, 22^. The step voltage is higher (50 mV) than the thermal energy of 26 meV ( = k_B_T/q) at 300 K. The I-V curves of staircase RESET using quantum conductance phenomenon have been simulated. The conductance is decreased by multiple of G_0_ in the interval of 50 meV (Fig. 10a), which is owing to generation of Ba^2+^ ions. To investigate the ionic states of the BaTiO_x_ SM, the EIS structure was fabricated (Fig. 2). The BaTiO_x_ sensing membrane shows good pH sensitivity of 48 mV/pH and linearity of 99.15% from pH values of 2 to 10 (Fig. 10b). Similar pH sensitivity values by using EIS structure are also reported with different sensing membranes such as 52.3 mV/pH for HfO_2_^44^, 56 mV/pH for TiO_2_^45^, 55 mV/pH for Gd_2_O_3_^46^, 56 mV/pH for Ta_2_O_5_^47^, 57.1 mV/pH for Al_2_O_3_^48^. Basically, the C-V curves are shifted towards positive direction with increasing pH value owing to deprotonation of BaTiO_x_ surface or adsorption of OH^−^ ions in the sensing membrane. On the other hand, the C-V curves are shifted towards negative direction owing to protonation (H^+^) of the BaTiO_x_ surface. Therefore, protonation/deprotonation^49^ of a sensing membrane shows pH sensitivity or reference voltage changes. The higher pH sensitivity of BaTiO_x_ membrane is observed than the bare SiO_2_ (48 vs. 35 mV/pH^50^) and comparable pH sensitivity (50 mV/pH) of TiO_2_ membrane (not shown here) owing to different oxidation states of Ba^+^ and Ba^2+^ ^42^. The oxidation-reduction (redox) of the sensing membrane is observed by measuring H_2_O_2_ and switching mechanism is explored as well. In contact of H_2_O_2_, the Ba surface is oxidized to Ba^2+^ (2BaO + O_2_ → 2BaO_2_^27^) or Ba changes to Ba^2+^ ions, and provides two electrons (e^−^) after H_2_O_2_ reduction. Possible surface reactions at Ba-sites are shown below.

4 $$B{a}^{o}\leftrightarrow B{a}^{2+}+2{e}^{-}$$

5 $${H}_{2}{O}_{2}\leftrightarrow O{H}^{-}+O{H}^{\ast }$$

6 $$O{H}^{\ast }+{e}^{-}\leftrightarrow O{H}^{-}$$

7 $$O{H}^{-}+{H}^{+}\leftrightarrow {H}_{2}O$$

From equations (4)–(7), the generation of Ba^2+^ ions increases with increasing H_2_O_2_ concentration from 1 nM to 1000 nM (Fig. 10c). By considering doping of 10^15^ cm^−3^ in p-Si, the work function is approximately 4.9 eV whereas the work function of Ba is 2.52–2.7 eV^42^. Droubay *et al*.^24^ have reported that the work function of BaO_2_ on Ag(001) is higher than the value of BaO (3.5 eV vs. 2.5 eV). This suggests that the work function of Ba increases after oxidation as well as the work function difference in between Si and BaO is reduced with increasing H_2_O_2_ concentration or Si band bending is reduced with H_2_O_2_ concentration. Due to work function modulation of BaO by oxidation/reduction, the reference voltage shifts towards positive direction with increasing H_2_O_2_ concentration as well as energy band bending of Si is decreased. Therefore, the reference voltage is needed to have flat-band of Si. This implies that the Ba^2+^ ion increases as well as work function increases with increasing the H_2_O_2_ concentration. On the other hand, the work function of Ti decreases after oxidation (Ф_Ti_ = 4.33 eV^42^ and Ф_TiO2_ = 4.13 eV^51^). Both TiO_2_ and SiO_2_ membrane do not show H_2_O_2_ sensing because of no oxidation/reduction of those membranes (Fig. 10c) due to strong bonding of Ti-O and Si-O. The reference voltage shift increases linearly with increasing H_2_O_2_ concentration from 10 nM to 1000 nM. However, pH value of 7 is unchanged during successive addition of H_2_O_2_. The BaTiO_x_ membrane detects also a low concentration of 1 nM with reference voltage shift of 6 mV, which is comparable with the reported H_2_O_2_ sensing of 2 to 10 nM by using different materials and methods^52–54^. From this H_2_O_2_ sensing, the oxidation of a single Ba atom confirms the evidences of the quantum conductance under RESET. The experimental and simulated I-V curves are given in Fig. 10d. The regions with different conduction mechanism are denoted by different colors in the simulated I-V curve. The parameters taken in the simulation i.e. the value of mobility of electron (μ = 2 × 10^4^ m^2^/V.s, close to reported value of 1 × 10^4^ m^2^/Vs^40^), density of state of conduction band (N_c_ = 8 × 10^24^ m^−3^, close to reported value of 8.5 × 10^24^/m^[3 40^), permittivity of BaTiO_3_ (ε_r_ = 85), hopping distance (a = 0.6 nm), frequency of thermal vibration (ν = 10^13^ Hz^41^), activation energy (E_A_ = 0.5 eV, close to the reported value of 0.8 eV^42^), and barrier height of 0.7 eV have been used in MATLAB simulator. Considering negative voltage modulated transport mechanisms and quantum conductance under staircase RESET, simulated and experimental I-V curves are filled well. This is very useful way to understand switching mechanism as well as multilevel operation of resistive switching phenomena. In addition, the S2 device shows excellent 1000 consecutive repeatable DC cycles, high speed (100 ns) program/erase endurance more than 10^7^ cycles (Fig. S2), and multi-level data retention at 85 °C (Fig. S3). The time-response of H_2_O_2_ sensing is also shown in Fig. S4, which promises repeatedly used of the sensor (supplementary information). This negative voltage modulated multi-level resistive switching with high resistance ratio and understanding of resistive switching mechanism of quantum conductance through H_2_O_2_ sensing will show a path towards solution for high density 3D multi-level cross-point memory application.

## Conclusion

In summary, the negative voltage modulated multi-level resistive switching and quantum conductance have been reported in simple Cr/BaTiO_x_/TiN structure. Amorphous nature of BaTiO_x_ switching material has been confirmed by the cross-sectional and plain view HRTEM. Different oxidation states of Ba are revealed from XPS. The switching characteristics follow the P-F and hopping conduction mechanism in HRS whereas Ohmic and hopping conduction have been observed in LRS. It is interesting to note that F-N conduction in very high field of negative cycle of HRS and quantum conductance in staircase RESET has been observed. For the realization of multi-level resistive switching, change in resistance with V_STOP_ voltage has been demonstrated and quantized by a parameter in the unit of mV/decade. The device with 2.5 nm thickness of BaTiO_x_ and 0.4 × 0.4 µm^2^ size exhibits a tunable, moderate change of HRS with stop voltage (217.39 mV/decade). By exploring the conduction mechanism of each HRS corresponding to each V_STOP_ voltage, the multi-level phenomenon has been explained through a gradual dissolution of oxygen vacancy filament. The devices also show current compliance dependent multi-level operation. Quantum conductance and multi-level switching phenomenon have been explained through evidence of H_2_O_2_ sensing mechanism. The BaTiO_x_ sensing membrane shows low concentration of H_2_O_2_ detection (1 nM) and good pH sensitivity of 48 mV/pH. The device with 2.5 nm thickness of BaTiO_x_ shows also high speed program/erase endurance of 10^7^ cycles with 100 ns pulse width and data retention of more than 3 hours at 85 °C. Oxygen migration based tunable multi-level RRAM with 2000 resistance ratio has been reported, which will be useful for high density 3-D cross-bar application. The explanation of switching mechanism using H_2_O_2_ sensing of the same material indicates the possibility of using memory and bio-sensor in bio-memory chip.

## Methods

### Memory device fabrication

At first, 8-inch Si wafer was cleaned by Radio Corporation of America (RCA) process. Then, 200 nm-thick SiO_2_ film was grown by thermal oxidation. A 160 nm-thick Ti as an adhesive layer and 40 nm-thick TiN as a bottom electrode (BE) were deposited on SiO_2_/Si substrate. Then, 150 nm-thick SiO_2_ as an insulating layer was deposited on BE. This SiO_2_ layer was patterned and etched out to create via-holes with size ranging from 0.4 × 0.4 µm^2^ to 4 × 4 µm^2^. The BaTiO_x_ as a switching material (SM) and 200 nm-thick Cr as a top electrode (TE) were deposited by rf sputtering. Both BaTiO_3_ and Cr targets were used. The deposition was done in the same sputtering system. Deposition parameters such as power, Ar flow rate, and deposition pressure were 100 W, 10 sccm, and 6 mTorr, respectively for both materials. The SMs with different thicknesses of 5 nm (S1) and 2.5 nm (S2) were deposited in a Cr/BaTiO_x_/TiN structure. Finally, lift-off process was performed to get the Cr/BaTiO_x_/TiN device, as shown in Fig. 1a. The electrical characteristics were obtained by applying bias on the TE, whereas the BE is connected to ground. Memory characteristics were measured using Agilent 4156C and Agilent B1500A semiconductor parameter analyzers.

### H_2_O_2_ sensor fabrication

To confirm the oxidation states of the BaTiO_x_ SM, the H_2_O_2_ sensing was performed by using electrolyte-insulator-semiconductor (EIS) structure. First, 4-inch p-type Si wafer was cleaned by RCA process. Then, the wafer was dipped into dilute HF solution and the SiO_2_ layer with a thickness of 40 nm was deposited by hot horizontal furnace at a temperature of 950 °C. Oxygen gas was used during growth of SiO_2_. Backside SiO_2_ layer was removed by using buffer oxide etching solution (BOE). Then, aluminum (Al) with a thickness of 200 nm was deposited on backside by thermal evaporation. Post-metal annealing (PMA) was performed in a hot horizontal furnace at a temperature of 450 °C for 10 min. Nitrogen gas with a flow rate of 2.5 SLM (standard liter per minute) was used during annealing. Then, the BaTiO_x_ sensing membrane with a thickness of approximately 2 nm was deposited on 40 nm-thick SiO_2_ layer by using the same deposition recipe of the SM. For comparison, the TiO_2_ and bare SiO_2_ membranes in EIS structure were also deposited. The sensing area was defined by lithography. Negative photo-resist, SU8 was used. The sensing area was 3.14 mm^2^. Then, one sensor was cut properly and fixed on Cu printed circuit board by using silver (Ag) paste. Epoxy was used to isolate in between Cu electrode and sensing area. Schematic view of a sensor is shown in Fig. 2. A reference electrode was used during capacitance-voltage (C–V) measurement in aqueous solutions with pH values from 2 to 10. The C-V characteristics with measurement frequency of 100 Hz were measured by using Agilent HP 4184 A *LCR* meter. The reference voltage was measured at capacitance (C) of 0.6C_ox_, where C_ox_ is accumulation capacitance.

### pH and H_2_O_2_ solution preparation

Sodium phosphate monobasic monohydrate (NaH_2_PO_4_.H_2_O), sodium phosphate dibasic anhydrous (Na_2_HPO_4_), sodium chloride (NaCl) were purchased from J. T. Baker Co. Ltd, (PA, USA) and hydrogen chloride (HCl) was purchased from Sigma-Aldrich (USA). Different pH buffer solutions (pH 2 to pH 10) were bought from Alfa- Aesar Co. Ltd (MA, USA). Hydrogen peroxide (H_2_O_2_, 31%) was purchased from BASF Co. Ltd. (Taipei, Taiwan). Detection of H_2_O_2_ was carried-out in phosphate buffer solution (PBS, 5 mM, pH 7) and the solution was prepared by adjusting the pH by 0.1 mol/l HCl in mixing appropriate amount of Na_2_HPO4 and NaH_2_PO_4_. To increase the ionic strength of solution, an amount of 4.6-gm of NaCl was dissolved in to buffer solution. The capacitance-voltage response was performed in 5 ml of PBS solution and further the concentration range of H_2_O_2_ from 1 nM to 1000 nM was increased by successive addition from stock solution of 1 µM H_2_O_2_. After preparation of each concentration of H_2_O_2_, pH value was checked by market available pH meter and the pH value of 7 was unchanged throughout H_2_O_2_ mixing in PBS solution. To obtain repeatable data, every concentration was measured by using three different sensors.

## Electronic supplementary material

Supplementary Infornation
