# Supplementary material for: Negative voltage modulated multi-level resistive switching by using a Cr/BaTiOx/TiN structure and quantum conductance through evidence of H2O2 sensing mechanism
Source: Sci Rep. 2017 Jul 5;7:4735. doi: 10.1038/s41598-017-05059-9 (PMC5498493; doi:10.1038/s41598-017-05059-9)
Supplement: Supplementary file 1 — Supplementary Infornation [file 41598_2017_5059_MOESM1_ESM.pdf]

Supplementary Information

Negative voltage modulated multi-level resistive switching by using a Cr/$BaTiO_x$/TiN structure and quantum conductance through evidence of H_2O_2 sensing mechanism

Somsubhra Chakrabarti[a], Sreekanth Ginnaram[a], Surajit Jana[a], Zong-Yi Wu[a], Kanishk Singh[a], Anisha Roy[a], Pankaj Kumar[a], Siddheswar Maikap[a,b,*], Jian-Tai Qiu[b], Hsin-Ming Cheng[c], Ling-Na Tsai[c], Ya-Ling Chang[d], Rajat Mahapatra[e], and Jer-Ren Yang[d]

[a]Thin Film Nano Tech. Lab., Department of Electronics Engineering, Chang Gung University, 259 Wen-Hwa 1st Rd., Kwei-Shan, Tao-Yuan, 33302, Taiwan

[b]Division of Gyn-Oncology, Department of Obs/Gyn, Chang Gung Memorial Hospital (CGMH), Tao-Yuan, 33302, Taiwan

[c]Material and Chemical Research Laboratories (MRL), Industrial Technology Research Institute (ITRI), Hsinchu, 310, Taiwan

[d]Department of Materials Science and Engineering, National Taiwan University, Taipei, 106, Taiwan

[e]Department of Electronics and Communications Engineering, National Institute of Technology (NIT), Durgapur, 713209, India

*Corresponding author: E-mail address: sidhu@mail.cgu.edu.tw Tel: +886-3-2118800 ext. 5785

<u>Thermal stability of 5 nm-thick $BaTiO_x$ film on SiO_2/Si substrate:</u>

The thermal stability of $BaTiO_x$ switching material (SM) has been investigated by annealing at 450°C, 600°C, and 750°C for 10 minutes in N_2 ambient. The 5 nm-thick $BaTiO_x$ film was deposited on 40 nm-thick SiO_2 film by RF sputtering. A p-type Si (100) substrate was used. The deposition parameters were kept the same as the switching material in RRAM devices. Three separate samples were annealed at 450°C, 600°C and 750°C. The XPS spectra are given in Fig.

S1. The $BaTiO_x$ film was analyzed by XPS using an Al ($K_\propto$) monochrome X-ray and at an energy of 1486.6 eV. The base vacuum pressure in the analytic chamber was 1×10^{-9} Torr. The analysis area was 650 μm in diameter. The Ba $3d_{5/2}$ and Ba$3d_{3/2}$ doublet spectra of all three annealed samples are fitted around 780.3 eV and 795.7 eV, respectively. Those peaks are almost the same with as-deposited sample (Fig. S1(a)). All Ba$3d_{5/2}$ peaks are de-convoluted into two peaks, where BaO peak is centered at 779.8 eV and BaO_2 peak is centered at 781.4 eV. The area

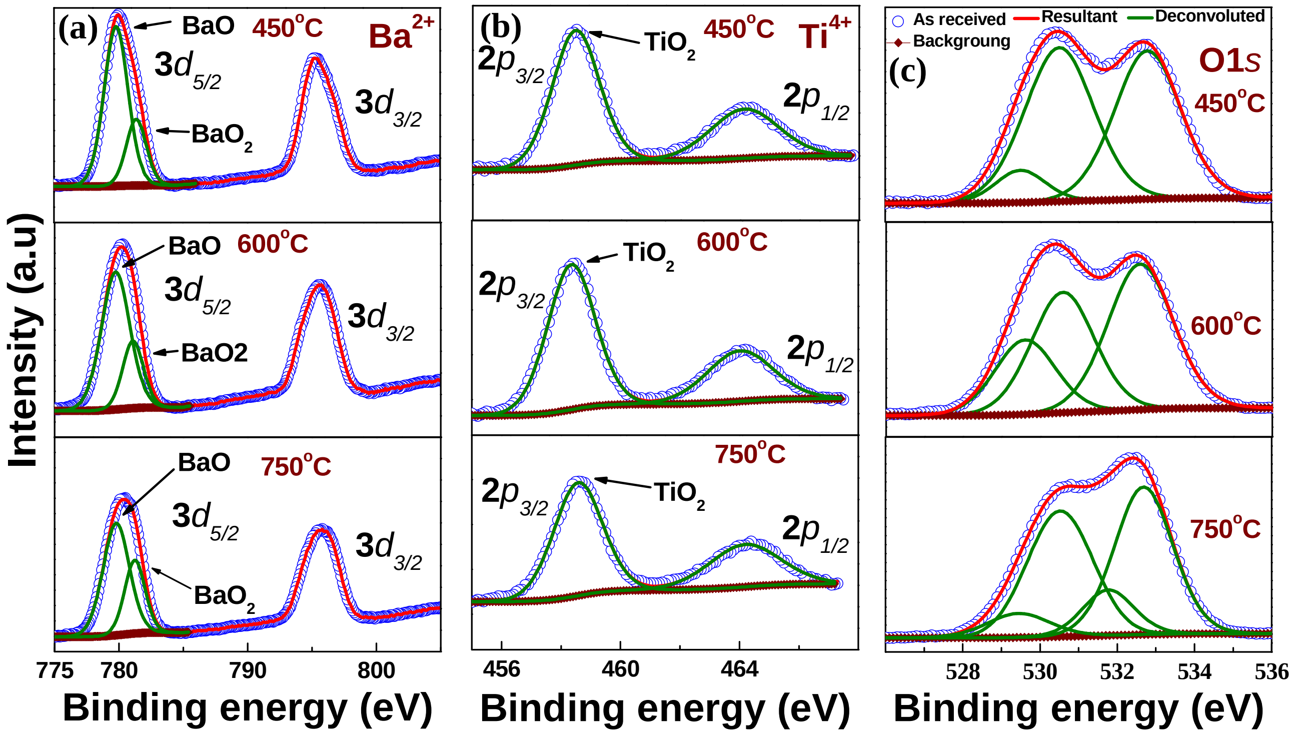

Fig. S1 XPS characteristics of (a) Ba3*d*, **(b)** Ti2*p* and **(c)** O1*s* spectra of the $BaTiO_x$ film with annealing temperature of 450°C, 600°C and 750°C in N_2 ambient.

ratios of BaO to BaO_2 for the annealing at 450°C, 600°C, and 750°C are found to be 2.88, 2.47, and 1.93, respectively. The BaO content is slightly decreased (or BaO_2 content is increased) with increasing annealing temperatures. However, there is negligible change of Ba oxidation state or binding energy, even at 750°C. Iqbal et al.[S1] have also reported the broadening of BaO_2 peak in $Ba_2YCu_3O_{7-\delta}$ film due to annealing at 900°C. Similarly, the Ti$2p_{3/2}$ peak is slightly shifted

towards higher binding energy of 0.5 eV as compared to the as-deposited films (458.5 eV vs. 458 eV), as shown in Fig. S1(b). Fig. S1(c) shows XPS spectra of O*1s*. The O*1s* spectra of the 450°C and 600°C annealed samples are de-convoluted into three peaks, where the 750°C annealed sample is de-convoluted into four peaks. In case of 750°C annealed sample, the fourth peak centered at 531.8 eV may be associated with nitrate/nitrite species. Vovk et al.[27] have reported similar peak owing to N_2 incorporation. The x value of the $BaTiO_x$ SM is found to be 1.69 up to 600°C annealing sample, which is negligible change as compared to the as-deposited film (x=1.98). However, the x value is decreased to 0.58 at 750°C. Saroukhani et al.[S2] have also reported that the Sr incorporated $BaTiO_3$ sample with a thickness of 500 nm becomes unstable after annealing at 800°C because the non-perovskite component in Ba3$d_{5/2}$ peak is increased extremely. In our case, we found that a thin (5 nm) $BaTiO_x$ film is thermally stable up to 600°C, which is acceptable for both resistive switching and bio-sensing.

Cyclic stability:

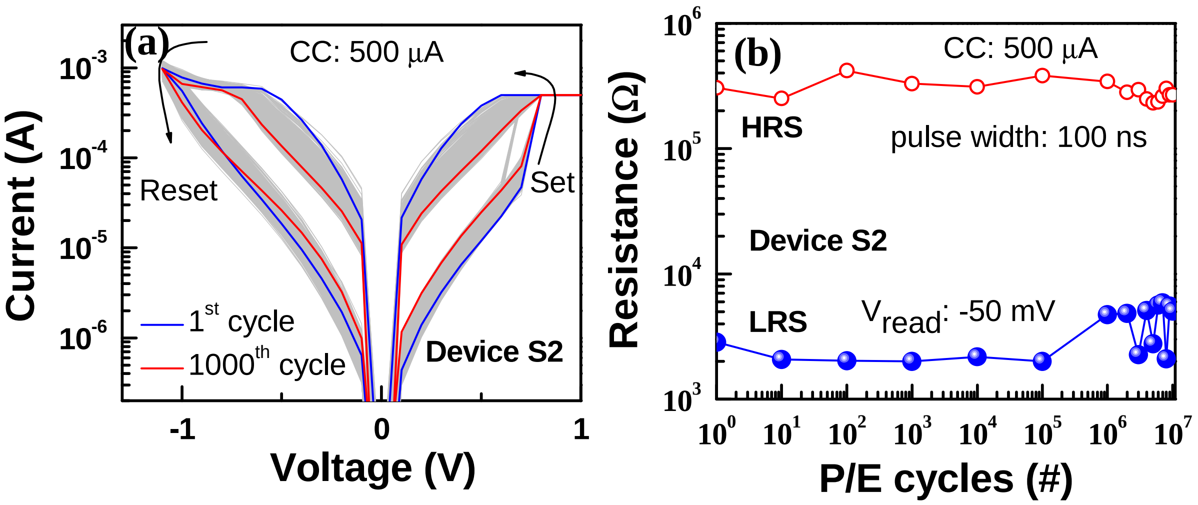

Fig. S2. (a) Excellent cyclic stability of 1000 repeatable cycles for the S2 devices at a current compliance of 500 μA. **(b)** Long program/erase (P/E) endurance up to 10^7 cycles with small pulse width of 100 ns. The P/E operation current was 500 μA.

Multi-level data retention at 85°C:

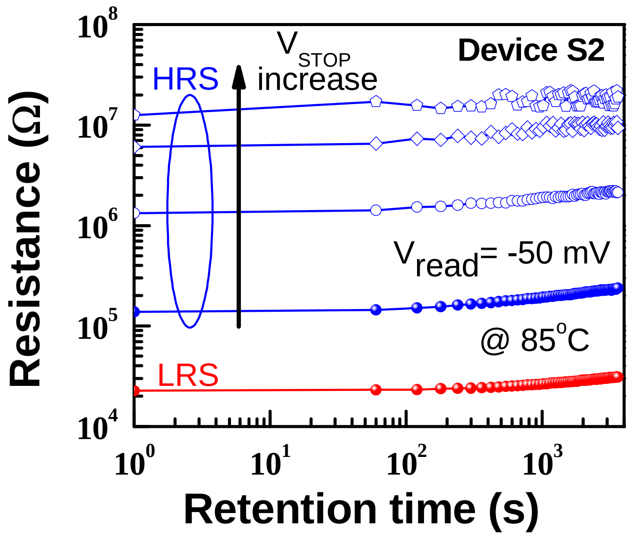

Fig. S3. Multi-level data retention is shown at 85°C for S2 devices. The current compliance was 500 μA. The LRS value at 85°C is increased than the value at room temperature because of metallic behavior of conducting filament, which is also reported by Chang et al.[S3] in Al/$BaTiO_3$/ITO resistive switching memory.

Time-response of $BaTiO_x$ membrane in EIS structure:

The time-response of H_2O_2 sensing by using $BaTiO_x$ membrane in electrolyte-insulation-semiconductor structure is given in Fig. S4. The reference voltage shift in PBS buffer solution is in noise level (3 mV shift). However, the reference voltage shift is increased rapidly up to ~15 mV by adding 10 nM H_2O_2 in PBS buffer solution. After washing in DI water, the sensor is dipped into PBS buffer solution, where the reference voltage shift is less than 3 mV. Therefore, this sensor can be used repeatedly.

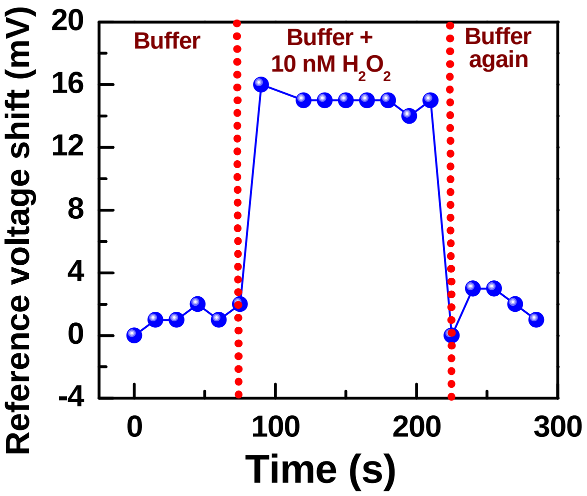

Fig. S4 Time-dependent H_2O_2 sensing. The sensor can be re-used after test.

<u>Reference:</u>

S1. Iqbal, Z. *et al.*, X-ray photo emission spectroscopy of the 90K superconductor $Ba_2YCu_3O_{7-\delta}$. *J. Mater. Res.* 2, 768 (1987).

S2. Saroukhani, Z., Tahmasebi, N., Mahdavi, S. M., & Nemati, A., Effect of working pressure and annealing temperature on microstructure and surface chemical composition of barium strontium titanate films grown by pulsed laser deposition. *Bull. Mater. Sci*. 38, 1645 (2015).

S3. Chang, Y. C., Xue, R. Y., & Wang, Y. H. Multilayered barium titanate thin films by sol-gel method for nonvolatile memory application. *IEEE Trans. Electron Devices*. 61, 4090 (2014).
